# Supplementary material for: Population-wide cerebellar growth models of children and adolescents
Source: Nat Commun. 2024 Mar 18;15:2351. doi: 10.1038/s41467-024-46398-2 (PMC10948906; doi:10.1038/s41467-024-46398-2)
Supplement: Supplementary file 1 — Supplementary Information [file 41467_2024_46398_MOESM1_ESM.pdf]

Supplementary Information for

"Population-wide Cerebellar Growth Models of Children and Adolescents"

Supplementary Figures

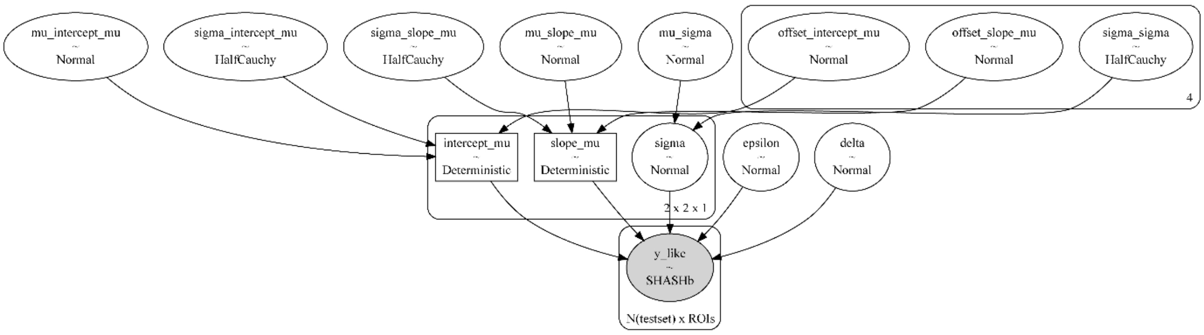

Supplementary Figure 1: Graphical representation of the normative model.
Hierarchical structure, model parameters and relationships between parameters are shown. Batch-effects are shown as 2x2 design (2 scanners x 2 sexes).

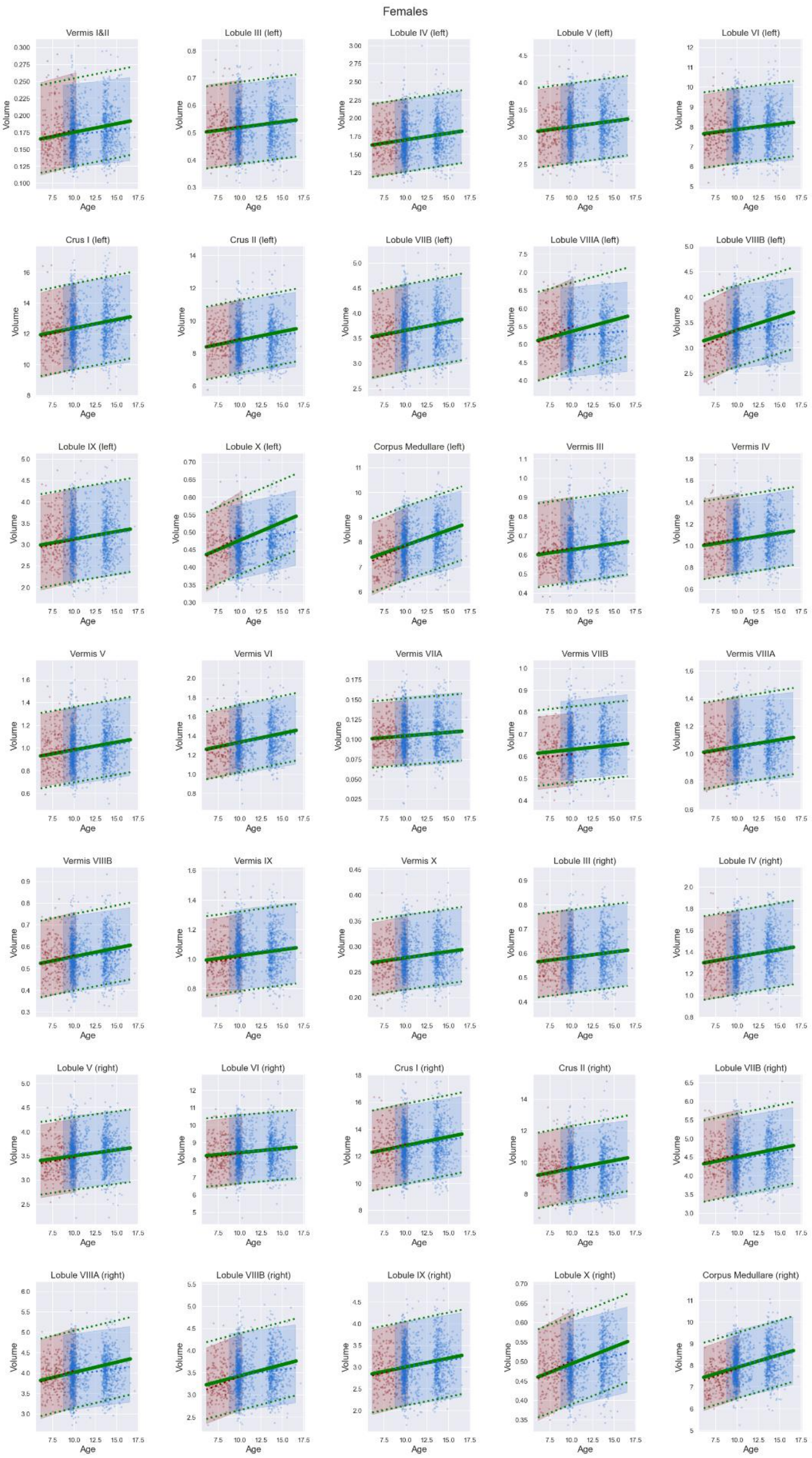

Supplementary Figure 2: Growth trajectory for each anatomical ROI for all females.
Bold green lines show the mean trajectory, dotted green lines represent what is within 2 standard deviations of the mean. In red all data points of females acquired on the first scanner (visit 1) are shown. Red dotted line and red shaded area illustrate the mean trajectory and what is within 2 standard deviations considering the batch-effect of the first scanner only. Analogous in blue, data points of females acquired on the second scanner (visit 2 & 3) are shown. Blue dotted line and blue shaded area illustrate the mean trajectory and what is within 2 standard deviations considering the batch-effect of the second scanner only. The y-axis shows volume in cubic centimeters (ccm). Source data are provided as a Source Data file.

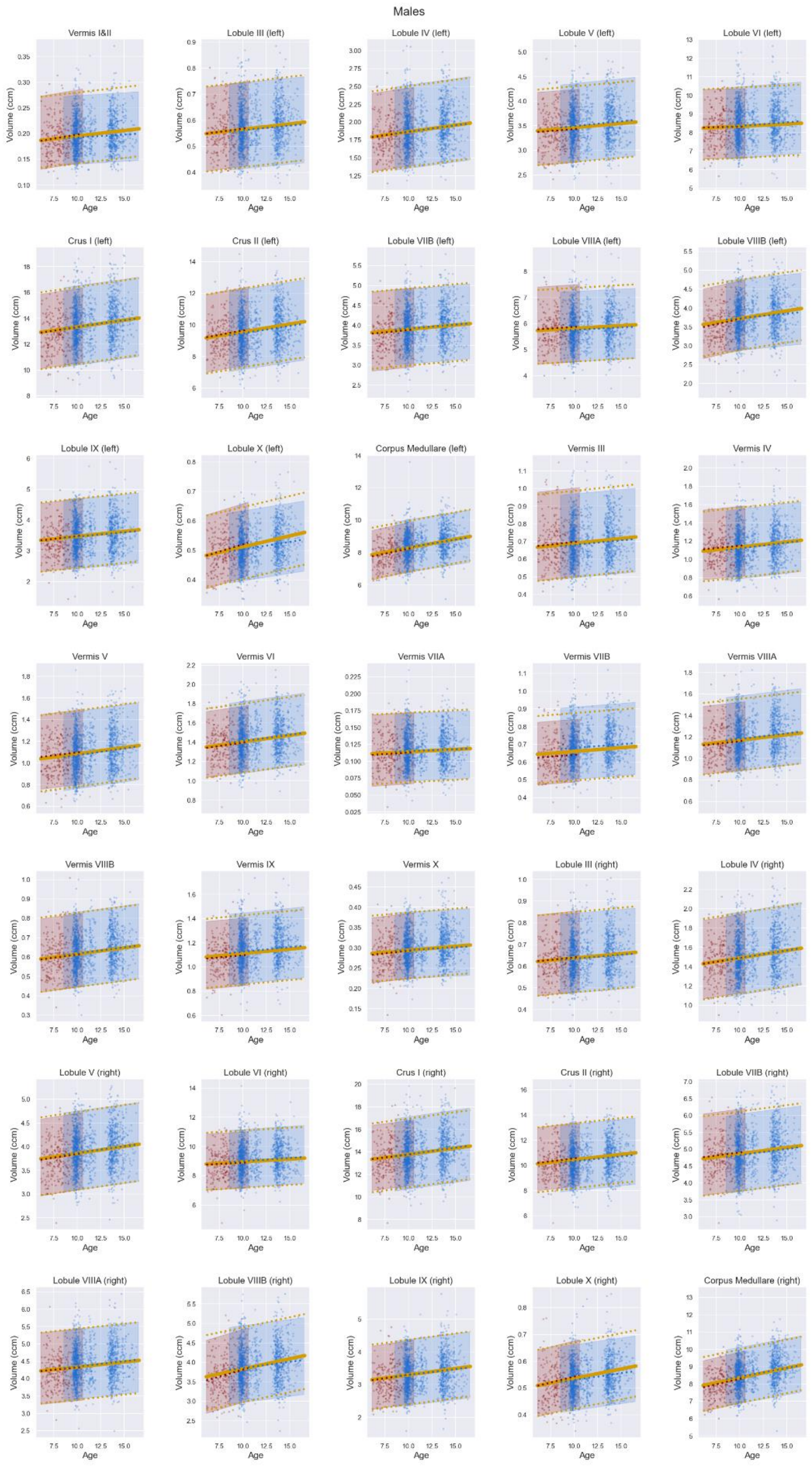

Supplementary Figure 3: Growth trajectory for each anatomical ROI for all males.
Bold yellow lines show the mean trajectory, dotted yellow lines represent what is within 2 standard deviations of the mean. In red all data points of males acquired on the first scanner (visit 1) are shown. Red dotted line and red shaded area illustrate the mean trajectory and what is within 2 standard deviations considering the batch-effect of the first scanner only. Analogous in blue, data points of males acquired on the second scanner (visit 2 & 3) are shown. Blue dotted line and blue shaded area illustrate the mean trajectory and what is within 2 standard deviations considering the batch-effect of the second scanner only. The y-axis shows volume in cubic centimeters (ccm). Source data are provided as a Source Data file.

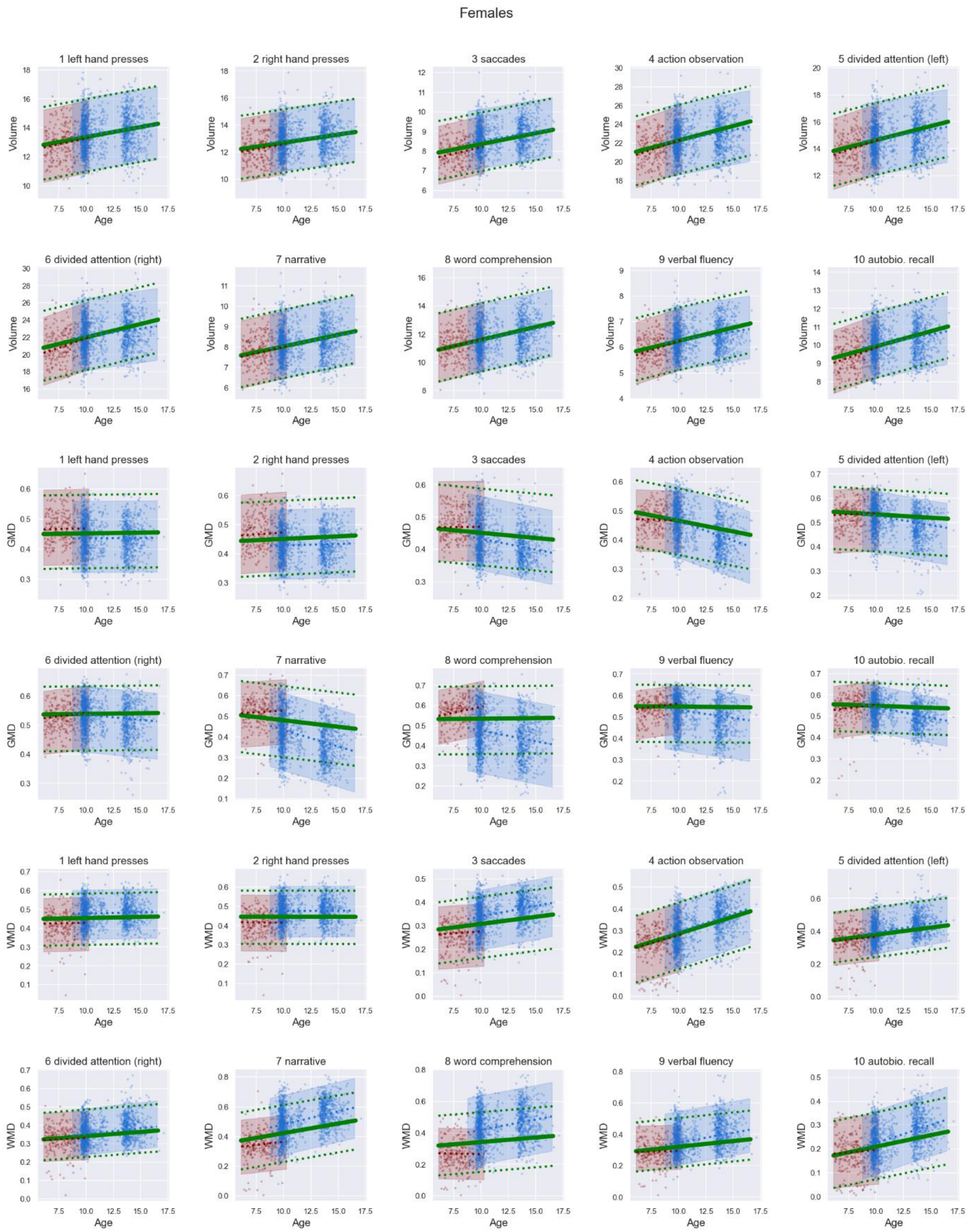

Supplementary Figure 4: Growth trajectory for each functional ROI for all females.

First 2 rows depict trajectories for volumes, 3rd and 4th row for GMD, 5th and 6th row for WMD. Bold green lines show the mean trajectory, dotted green lines represent what is within 2 standard deviations of the mean. In red all data points of females acquired on the first scanner (visit 1) are shown. Red dotted line and red shaded area illustrate the mean trajectory and what is within 2 standard deviations considering the batch-effect of the first scanner only. Analogous in blue, data points of females acquired on the second scanner (visit 2 & 3) are shown. Blue dotted line and blue shaded area illustrate the mean trajectory and what is within 2 standard deviations considering the batch-effect of the second scanner only. Source data are provided as a Source Data file.

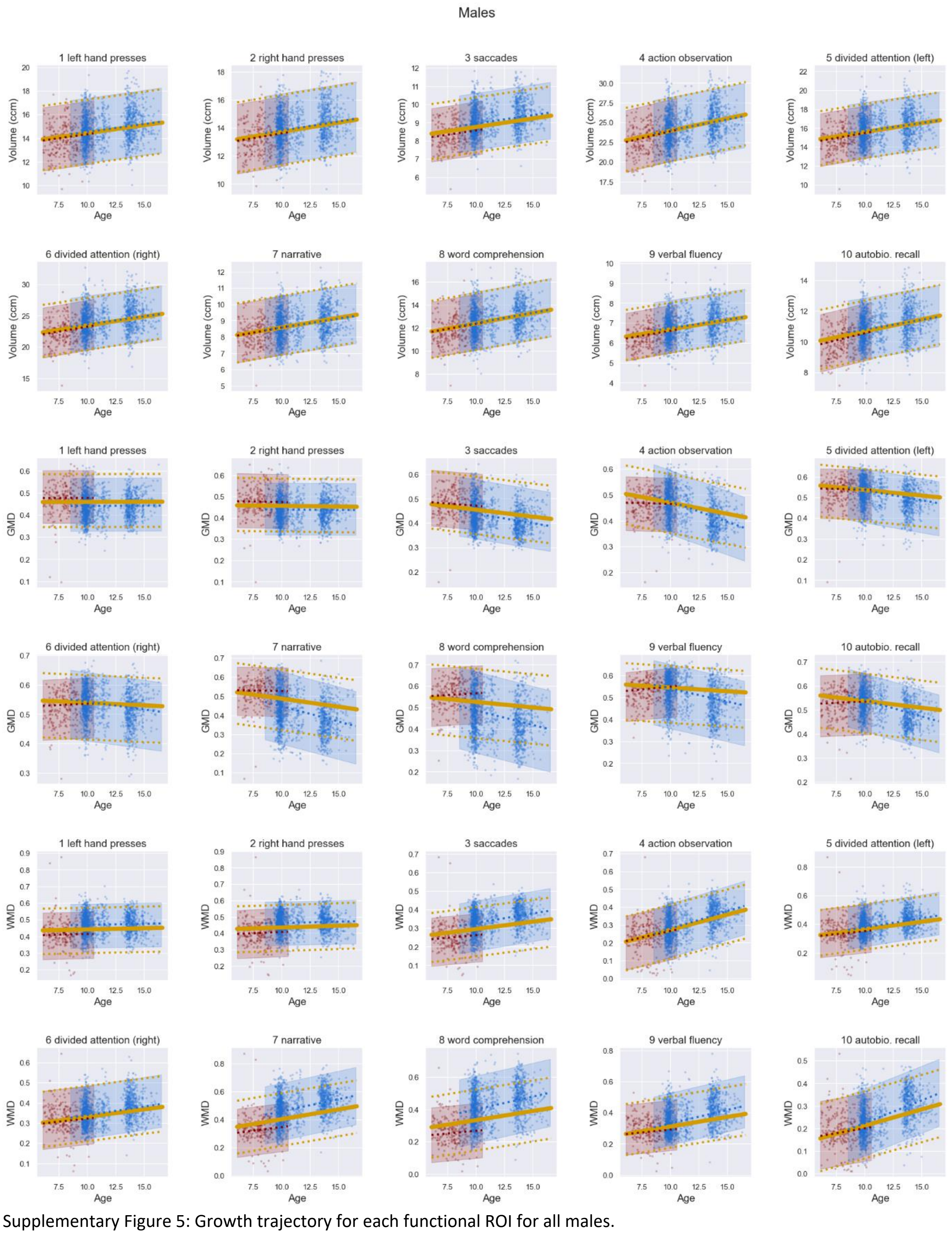

Supplementary Figure 5: Growth trajectory for each functional ROI for all males.

First 2 rows depict trajectories for volumes, 3rd and 4th row for GMD, 5th and 6th row for WMD. Bold yellow lines show the mean trajectory, dotted yellow lines represent what is within 2 standard deviations of the mean. In red all data points of males acquired on the first scanner (visit 1) are shown. Red dotted line and red shaded area illustrate the mean trajectory and what is within 2 standard deviations considering the batch-effect of the first scanner only. Analogous in blue, data points of males acquired on the second scanner (visit 2 & 3) are shown. Blue dotted line and blue shaded area illustrate the mean trajectory and what is within 2 standard deviations considering the batch-effect of the second scanner only. Source data are provided as a Source Data file.

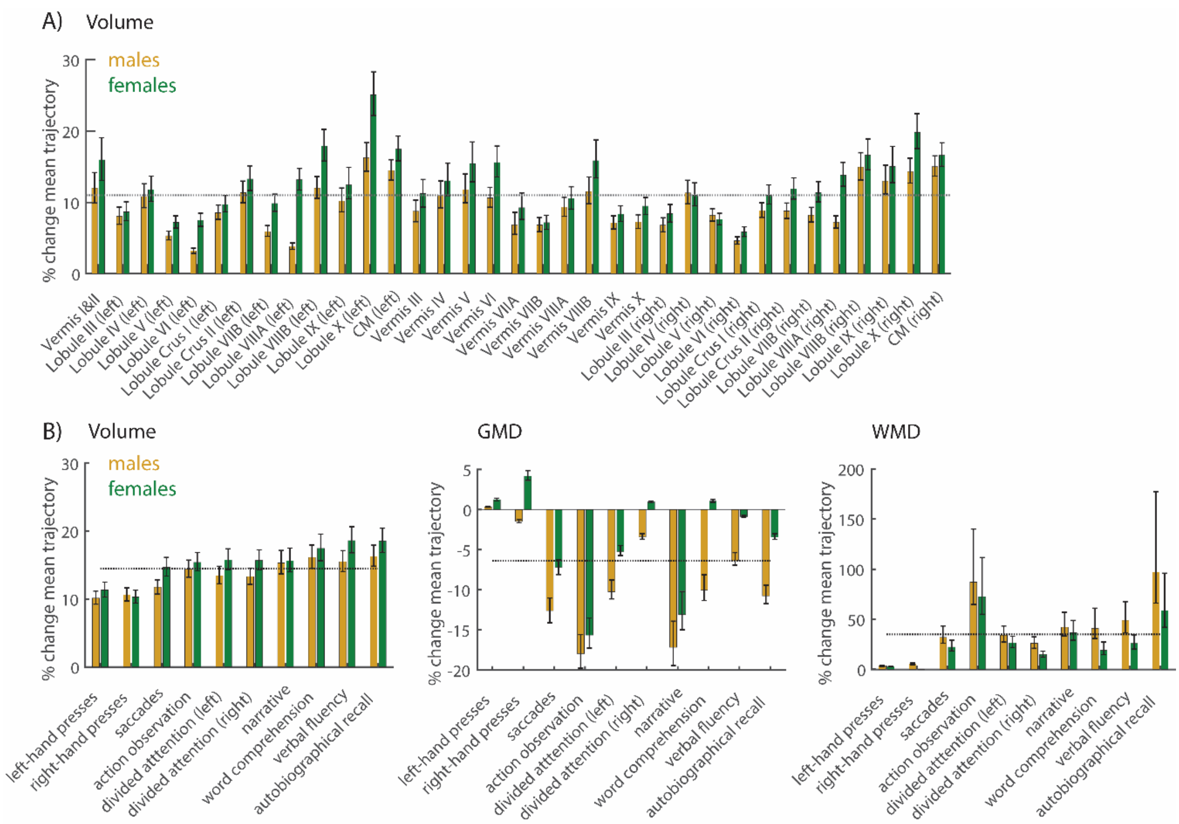

Supplementary Figure 6: Percentage change of the mean linear trajectory of males and females between the ages of 6 and 17 for the A) volume in the anatomical and B) volume, *Grey Matter Density* (GMD), and *White Matter Density* (WMD) in the functional parcellation.

Horizontal lines depict the mean percentage change over ROIs, error bars represent percentage change with +/- 1 standard deviation of the mean trajectory. Important to note: percentage change is highly sensitive to initial values. The more extreme the initial value, the more likely is a high percentage change (e.g.: WMD autobiographical recall where initial values are very low (see Supplementary Figures 4 & 5)). Source data are provided as a Source Data file.

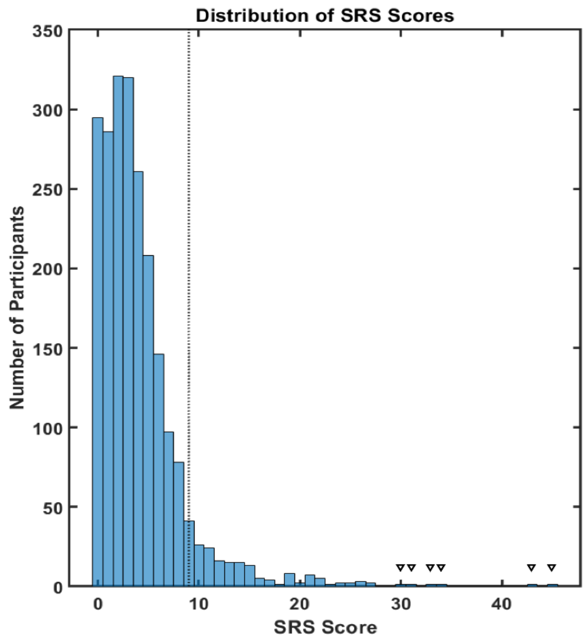

Supplementary Figure 7: Distribution of *Social Responsiveness Scale* (SRS) scores in the Generation R cohort (n=2,210; participants without SRS information excluded).

The dotted line illustrates the 90th percentile (raw score >= 9 [n=198]), triangles draw attention to high SRS scores of single participants. Source data are provided as a Source Data file.

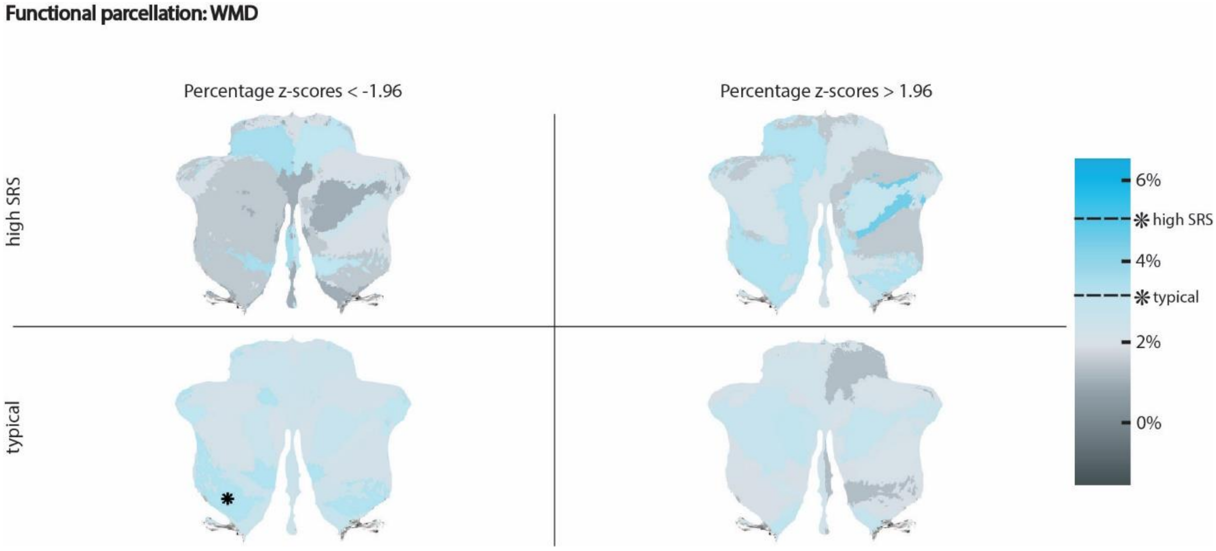

Supplementary Figure 8: Percentage of individuals with large negative (z-score < -1.96) and large positive (z-score > 1.96) deviations in *White Matter Density* (WMD) in functional ROIs.
Asterisks indicate ROIs in which children with high SRS and children with typical SRS scores have a significantly higher percentages of large deviation than expected (typical > 3.13%, high SRS > 5.05%; binomial test, p<0.05). Top row: children with high SRS scores, likely to fall on the Autism spectrum. Bottom row: typically developing children. Source data are provided as a Source Data file.

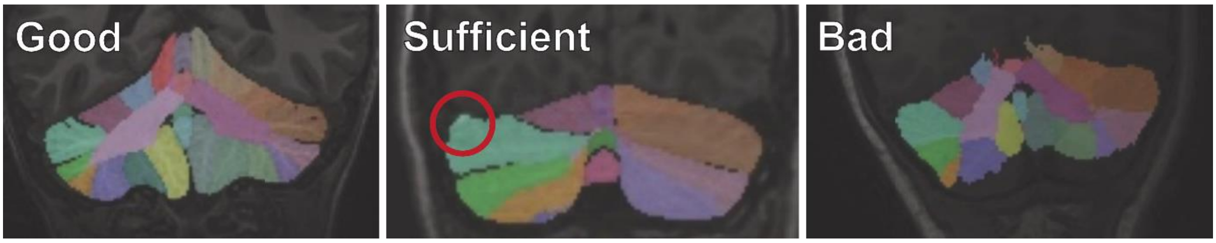

Supplementary Figure 9: Visual examples of segmented scans rated as either good, sufficient, or bad.
Inaccuracies of only a few voxels were rated as sufficient (e.g., vasculature was captured marked by red circle), whereas marked inaccuracies throughout several parcels were rated as bad and therefore excluded from analysis.

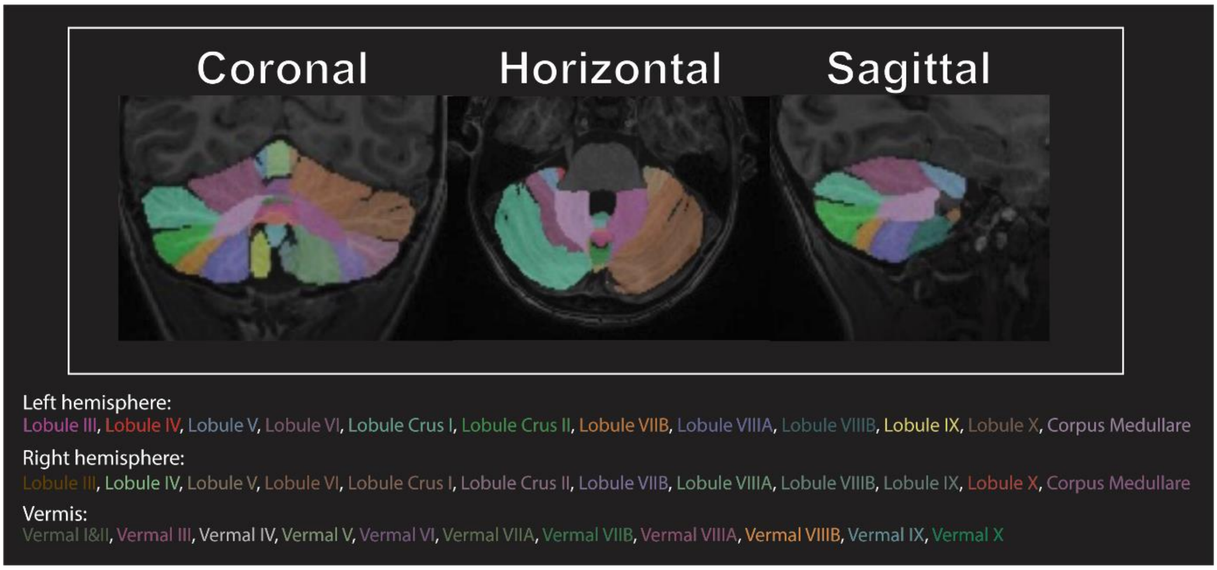

Supplementary Figure 10: Example scan that was automatically segmented in anatomical ROIs using the MAGeT algorithm. Labels for each ROI in their respective colors are shown. The MAGeT algorithm subdivides the cerebellum into 11 vermal and 22 hemispheric lobules (11 on each hemisphere). Additionally, the central white matter, the corpus medullare, is segmented in each hemisphere. White matter that extends into the folia of the lobules was segmented as part of the lobules.

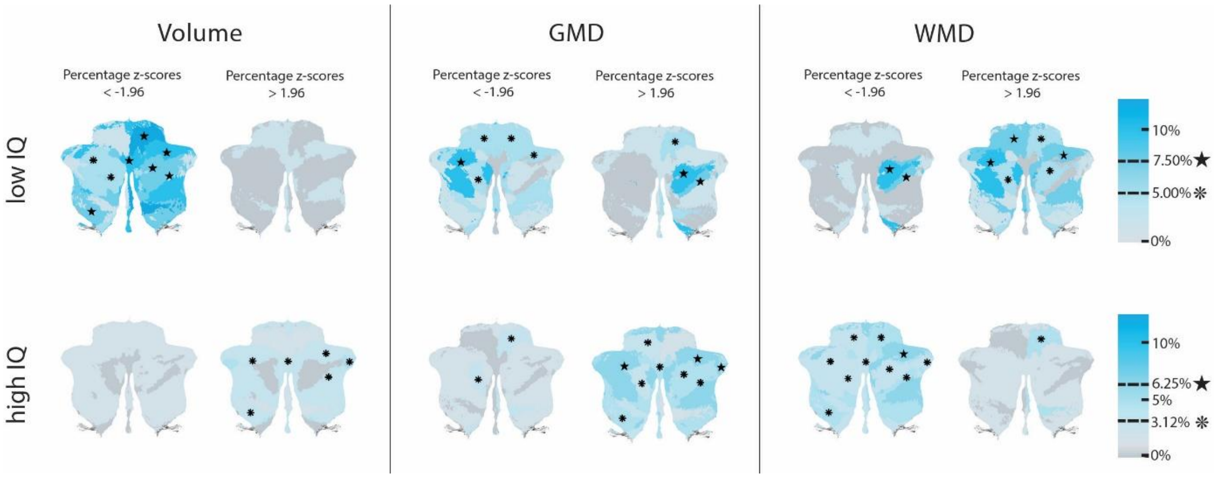

Supplementary Figure 11: Large deviations in functional ROIs stratified by IQ.

Percentage of individuals with large negative (z-score < -1.96) and large positive (z-score > 1.96) deviations in volume, *Grey Matter Density* (GMD), and *White Matter Density* (WMD) in functional ROIs is shown. Results are stratified in low (<70 [n=40]) and high IQ (>130 [n=64]). As sample sizes differ between IQ groups, and thus expected proportions of extreme deviations under the null hypothesis differ as well, significance of the percentage of children with large deviations at the $p = 0.05$ and $p = 0.01$ level were evaluated using Binomial testing (observed vs. expected number of participants with $z > 1.96$ / $z < -1.96$ in low and high IQ children, given a null hypothesized probability of $p0 = 0.025$, one-sided). Asterisks ($p<0.05$) and stars ($p<0.01$) indicate ROIs in which children have a significantly higher percentages of large deviation than expected (low IQ > 5.00% ($p<0.05$) and > 7.50% ($p<0.01$), high IQ > 3.12% ($p<0.05$) and > 6.25% ($p<0.01$)). Children in the low IQ group present with lower volumes than expected throughout several ROIs particularly on the right hemisphere (negative deviations at $p<0.01$ level: 2 Right-hand (motor) presses, 3 Saccades, 4 Action observation, 6 Divided attention (right hemisphere), 8 Word comprehension, and 9 Verbal fluency). Lower IQ was further associated with more negative as well as positive deviations in GMD (negative deviations at $p<0.01$ level: 5 Divided attention (left hemisphere); positive deviations at p<0.01 level: 8 Word comprehension, and 9 Verbal fluency) and WMD (negative deviations at $p<0.01$ level: 8 Word comprehension, and 9 Verbal fluency; positive deviations at $p<0.01$ level: 1 Left-hand (motor) presses, 5 Divided attention (left hemisphere), 6 Divided attention (right hemisphere)) specifically in posterior ROIs relating to cognitive function. Interestingly, high deviations in the same ROIs can be seen in the low and high IQ group in GMD and WMD. This might relate to non-linear effects of IQ on brain structure. Source data are provided as a Source Data file.

Supplementary Tables

Supplementary Table 1: sample characteristics of the training and test sets

<table>
<tr><th></th><th colspan="3">Training set
n=3,689</th><th colspan="3">Test set
n=3,581</th><th>Total
n=7,270</th></tr>
<tr><th></th><th>Visit 1
n=496</th><th>Visit 2
n=1,961</th><th>Visit 3
n=1,319</th><th>Visit 1
n=483</th><th>Visit 2
n=1,968</th><th>Visit 3
n=1,224</th><th></th></tr>
<tr><td>Age
Mean [Range]</td><td>7.9 [6.1 – 10.7]</td><td>10.1 [8.7 – 12.0]</td><td>14.0 [12.7 – 16.6]</td><td>7.9 [6.2 – 10.7]</td><td>10.1 [8.6 – 12.0]</td><td>14.0 [12.6 – 17.1]</td><td>11.2 [6.1 – 17.1]</td></tr>
<tr><td>Sex
(M|F)</td><td>53.8% | 46.2%</td><td>48.1% | 51.9%</td><td>47.6% | 52.4%</td><td>50.9% | 49.1%</td><td>51.2% | 48.8%</td><td>48.2% | 51.8%</td><td>49.4% | 50.6%</td></tr>
<tr><td>Income
(Low | Medium | High)</td><td>4.9% | 38.3% | 47.9%</td><td>6.0% | 31.2% | 49.8%</td><td>4.5% | 34.3% | 49.7%</td><td>7.7% | 38.9% | 45.9%</td><td>4.8% | 34.1% | 49.1%</td><td>5.0% | 33.2% | 47.7%</td><td>5.3% | 33.8% | 48.9%</td></tr>
<tr><td>Education Mother
(Low | High)</td><td>39.6% | 60.4%</td><td>33.5% | 66.5%</td><td>34.1% | 65.9%</td><td>42.2% | 57.8%</td><td>34.9% | 65.1%</td><td>35.6% | 64.4%</td><td>35.3% | 64.7%</td></tr>
<tr><td>IQ
(Low | Medium | High)</td><td>12.2% | 59.8% | 15.6%</td><td>9.1% | 56.9% | 15.8%</td><td>8.5% | 57.7% | 17.2%</td><td>11.4% | 60.3% | 16.2%</td><td>10.1% | 53.7% | 18.3%</td><td>10.6% | 55.7% | 15.5%</td><td>9.8% | 56.4% | 16.7%</td></tr>
<tr><td>Ethnicity
(Dutch | European (Non-Dutch) | Non-European)</td><td>68.0% | 6.5% | 25.6%</td><td>59.6% | 8.5% | 30.4%</td><td>60.0% | 8.3% | 29.5%</td><td>67.8% | 6.7% | 25.6%</td><td>58.2% | 8.4% | 30.9%</td><td>57.6% | 9.2% | 30.9%</td><td>60.1% | 8.3% | 29.8%</td></tr>
<tr><td>CBCL
(Low | High)</td><td>58.6% | 22.9%</td><td>67.1% | 17.1%</td><td>64.3% | 17.0%</td><td>57.6% | 19.3%</td><td>67.7% | 16.9%</td><td>65.0% | 14.3%</td><td>65.2% | 17.1%</td></tr>
</table>

* percentages do not always add up to 100% due to missing data from some participants

ROI	linear<br>loo [SE]	bspline<br>loo [SE]	difference [SE of difference]
Vermis I & II	-4910.89 [45.54]	-4911.74 [45.45]	0.85 [2.58]
Lobule III (left)	-5034.17 [43.25]	-5036.97 [43.29]	2.79 [2.51]
Lobule IV (left)	-4966.64 [44.45]	-4984.73 [44.56]	2.10 [2.45]
Lobule V (left)	-4940.60 [42.93]	-4945.15 [43.04]	4.55 [1.95]
Lobule VI (left)	-5078.43 [45.37]	-5082.04 [45.35]	3.61 [1.73]
Lobule Crus 1 (left)	-4992.30 [43.99]	-4995.34 [43.96]	3.04 [1.67]
Lobule Crus 2 (left)	-4981.02 [45.78]	-4980.35 [45.78]	0.67 [3.10]
Lobule VIIB (left)	-5066.31 [44.01]	-5069.97 [44.05]	3.66 [1.88]
Lobule VIIIA (left)	-4915.24 [45.22]	-4914.21 [45.11]	1.03 [3.25]
Lobule VIIIB (left)	-4800.77 [45.32]	-4799.91 [45.44]	0.86 [3.30]
Lobule IX (left)	-5040.51 [46.02]	-5042.22 [46.03]	1.72 [2.75]
Lobule X (left)	-4914.25 [45.01]	-4913.38 [44.96]	0.87 [3.47]
Corpus medullare (left)	-4929.63 [45.65]	-4925.68 [45.68]	3.95 [3.79]
Vermis III	-4961.70 [46.54]	-4965.54 [46.48]	3.84 [1.89]
Vermis IV	-5067.51 [45.57]	-5072.15 [45.20]	4.64 [1.43]
Vermis V	-5005.63 [42.50]	-5009.64 [42.44]	4.01 [1.68]
Vermis VI	-5060.31 [44.72]	-5063.01 [44.90]	2.71 [2.31]
Vermis VIIA	-5084.65 [47.27]	-5089.64 [47.14]	4.99 [1.63]
Vermis VIIB	-5050.23 [44.62]	-5056.92 [44.61]	6.70 [1.10]
Vermis VIIIA	-4840.80 [45.91]	-4843.85 [45.90]	3.06 [2.24]
Vermis VIIIB	-4916.88 [43.85]	-4920.07 [43.98]	3.19 [2.13]
Vermis IX	-4994.13 [45.09]	-4997.92 [45.06]	3.79 [2.15]
Vermis X	-5056.58 [48.49]	-5058.67 [48.53]	2.10 [2.17]
Lobule III (right)	-5017.72 [45.46]	-5020.44 [45.58]	2.72 [2.00]
Lobule IV (right)	-4966.79 [43.61]	-4968.72 [43.56]	1.93 [2.13]
Lobule V (right)	-4835.30 [45.11]	-4838.61 [45.17]	3.31 [1.99]
Lobule VI (right)	-5067.93 [45.63]	-5072.41 [45.72]	4.48 [1.41]
Lobule Crus 1 (right)	-4974.52 [43.50]	-4977.94 [43.50]	3.42 [1.90]
Lobule Crus 2 (right)	-4970.96 [44.83]	-4972.03 [44.79]	1.07 [2.73]
Lobule VIIB (right)	-4997.19 [43.77]	-5001.44 [43.69]	4.25 [2.10]
Lobule VIIIA (right)	-4920.04 [45.58]	-4921.01 [45.61]	0.97 [2.76]
Lobule VIIIB (right)	-4778.78 [45.31]	-4781.65 [45.30]	2.87 [2.44]
Lobule IX (right)	-5018.76 [44.65]	-5018.51 [44.57]	0.25 [3.17]
Lobule X (right)	-4905.17 [43.62]	-4908.13 [43.62]	2.96 [2.34]
Corpus medullare (right)	-4892.49 [46.78]	-4892.19 [46.79]	0.30 [3.29]
left hand presses (Volume)	-4814.67 [44.80]	-4818.54 [44.91]	3.87 [2.11]
right hand presses (Volume)	-4783.62 [45.92]	-4786.92 [46.00]	3.30 [2.13]
saccades (Volume)	-4822.83 [44.19]	-4825.58 [44.40]	2.75 [2.40]
action observation (Volume)	-4708.00 [44.40]	-4709.21 [44.45]	1.22 [2.73]
divided attention (left) (Volume)	-4838.92 [44.82]	-4838.44 [44.80]	0.47 [3.15]
divided attention (right) (Volume)	-4808.71 [46.02]	-4811.82 [46.02]	3.11 [2.00]
narrative (Volume)	-4924.14 [42.77]	-4923.69 [42.69]	0.45 [3.35]
word comprehension (Volume)	-4833.15 [43.01]	-4834.34 [43.07]	1.19 [2.62]
verbal fluency (Volume)	-4776.38 [44.56]	-4778.28 [44.60]	1.91 [2.68]
autobiographical recall (Volume)	-4728.35 [44.27]	-4729.09 [44.36]	0.74 [2.80]
left hand presses (GMD)	-5166.46 [44.01]	-5156.58 [44.39]	9.87 [5.50]
right hand presses (GMD)	-5147.05 [45.60]	-5142.16 [45.83]	4.89 [4.03]
saccades (GMD)	-4930.29 [48.30]	-4920.94 [48.40]	9.34 [4.39]
action observation (GMD)	-4857.93 [43.38]	-4845.04 [43.32]	12.89 [6.43]
divided attention (left) (GMD)	-4998.79 [46.76]	-4989.39 [46.91]	9.40 [5.10]
divided attention (right) (GMD)	-5135.83 [45.19]	-5130.88 [45.38]	4.95 [4.15]
narrative (GMD)	-4743.98 [45.23]	-4729.98 [45.35]	14.00 [5.59]
word comprehension (GMD)	-4840.82 [48.20]	-4832.78 [48.15]	8.05 [5.07]
verbal fluency (GMD)	-4882.19 [46.91]	-4876.16 [47.06]	6.03 [5.09]
autobiographical recall (GMD)	-4939.08 [48.31]	-4916.27 [48.08]	22.81 [7.89]
left hand presses (WMD)	-5067.61 [47.37]	-5055.36 [47.57]	12.25 [5.83]
right hand presses (WMD)	-5037.57 [48.02]	-5031.54 [48.34]	6.02 [4.45]
saccades (WMD)	-4717.58 [50.32]	-4702.22 [50.21]	15.35 [5.66]
action observation (WMD)	-4707.40 [43.81]	-4695.48 [44.00]	11.92 [5.74]
divided attention (left) (WMD)	-4858.86 [49.10]	-4844.21 [49.28]	14.65 [5.85]
divided attention (right) (WMD)	-5008.70 [47.83]	-5000.85 [48.34]	7.85 [4.94]
narrative (WMD)	-4556.83 [46.12]	-4541.50 [46.25]	15.33 [5.74]
word comprehension (WMD)	-4604.72 [48.00]	-4595.59 [47.95]	9.14 [5.11]
verbal fluency (WMD)	-4756.39 [46.60]	-4744.21 [46.94]	12.18 [5.85]
autobiographical recall (WMD)	-4696.75 [45.18]	-4665.38 [45.19]	31.37 [8.81]

Supplementary Table 2: *Leave-one-out* (LOO) Cross Validation. LOO, *standard error* (SE) for the LOO computations of linear and b-spline models are shown as well for difference in LOO and the SE of the difference for all anatomical and functional ROIs.

Anatomical lobular ROIS	Volume<br>Mean standardized β Age [95%CI Mean]<br>Left hemisphere	| Right hemisphere	Anatomical vermal ROIs	Volume<br>Mean standardized β Age [95%CI Mean]
			Vermis I&II<br>MALES<br>FEMALES	 0.142 [1.140 – 1.44]<br>0.167 [0.165 – 0.169]
Lobule III<br>MALES<br>FEMALES	 0.116 [0.115 – 0.117]<br>0.115 [0.114 – 0.116]	 0.100 [0.099 - 0.100]<br>0.112 [0.111 - 0.113]	Vermis III<br>MALES<br>FEMALES	 0.107 [0.106 - 0.108]<br>0.124 [0.123 - 0.124]
Lobule IV<br>MALES<br>FEMALES	 0.148 [0.147 – 0.149]<br>0.146 [0.146 – 0.147]	 0.162 [0.162 - 0.163]<br>0.144 [0.143 - 0.145]	Vermis IV<br>MALES<br>FEMALES	 0.138 [0.137 - 0.139]<br>0.151 [0.150 - 0.152]
Lobule V<br>MALES<br>FEMALES	 0.095 [0.094 – 0.096]<br>0.117 [0.116 – 0.118]	 0.154 [0.153 - 0.155]<br>0.130 [0.129 - 0.132]	Vermis V<br>MALES<br>FEMALES	 0.144 [0.143 - 0.145]<br>0.169 [0.168 - 0.170]
Lobule VI<br>MALES<br>FEMALES	 0.058 [0.056 – 0.059]<br>0.126 [0.124 – 0.128]	 0.086 [0.085 - 0.087]<br>0.102 [0.101 - 0.104]	Vermis VI<br>MALES<br>FEMALES	 0.165 [0.164 - 0.166]<br>0.226 [0.224 - 0.228]
Crus I<br>MALES<br>FEMALES	 0.153 [0.152 – 0.154]<br>0.161 [0.160 – 0.162]	 0.156 [0.155 - 0.157]<br>0.179 [0.177 - 0.180]	Vermis VIIA<br>MALES<br>FEMALES	 0.069 [0.068 - 0.070]<br>0.085 [0.084 - 0.086]
Crus II<br>MALES<br>FEMALES	 0.178 [0.177 – 0.179]<br>0.191 [0.189 – 0.192]	 0.145 [0.144 - 0.146]<br>0.179 [0.177 - 0.180]		
Lobule VIIB<br>MALES<br>FEMALES	 0.104 [0.103 – 0.105]<br>0.160 [0.158 – 0.161]	 0.142 [0.141 - 0.143]<br>0.181 [0.179 - 0.182]	Vermis VIIB<br>MALES<br>FEMALES	 0.098 [0.097 - 0.098]<br>0.098 [0.097 - 0.098]
Lobule VIIIA<br>MALES<br>FEMALES	 0.069 [0.067 – 0.070]<br>0.210 [0.207 – 0.213]	 0.126 [0.125 - 0.128]<br>0.219 [0.217 - 0.222]	Vermis VIIIA<br>MALES<br>FEMALES	 0.129 [0.128 - 0.130]<br>0.130 [0.129 - 0.132]
Lobule VIIIB<br>MALES<br>FEMALES	 0.192 [0.190 – 0.194]<br>0.252 [0.249 – 0.255]	 0.229 [0.227 - 0.231]<br>0.227 [0.224 - 0.229]	Vermis VIIIB<br>MALES<br>FEMALES	 0.149 [0.147 - 0.150]<br>0.181 [0.180 - 0.183]
Lobule IX<br>MALES<br>FEMALES	 0.129 [0.127 - 0.130]<br>0.141 [0.139 - 0.142]	 0.171 [0.170 - 0.172]<br>0.179 [0.177 - 0.180]	Vermis IX<br>MALES<br>FEMALES	 0.113 [0.112 - 0.114]<br>0.121 [0.121 - 0.122]
Lobule X<br>MALES<br>FEMALES	 0.280 [0.277 - 0.282]<br>0.390 [0.386 - 0.394]	 0.250 [0.248 - 0.251]<br>0.312 [0.310 - 0.315]	Vermis X<br>MALES<br>FEMALES	 0.115 [0.113 - 0.116]<br>0.140 [0.139 - 0.142]
Corpus medullare<br>MALES<br>FEMALES	 0.293 [0.292 -0.295]<br>0.334 [0.331 - 0.336]	 0.303 [0.301 - 0.304]<br>0.315 [0.313 - 0.317]		

Supplementary Table 3: Mean standardized age β coefficients (slopes) and 95% *confidence interval* (CI) of the mean for all anatomical ROIs stratified by sex. While all slopes are significantly different from 0, some effects (standardized coefficients) are small.

FUNCTIONAL REGIONS	VOLUME<br><br>MEAN STANDARDIZED Β AGE [95%CI MEAN]	GMD<br><br>MEAN STANDARDIZED Β AGE [95%CI MEAN]	WMD<br><br>MEAN STANDARDIZED Β AGE [95%CI MEAN]
1 LEFT-HAND PRESSES			
Males	0.211 [0.210 – 0.212]	0.005 [0.005 - 0.006]	0.047 [0.046 - 0.048]
Females	0.217 [0.216 – 0.218]	0.020 [0.019 - 0.021]	0.039 [0.038 - 0.040]
2 RIGHT-HAND PRESSES			
Males	0.227 [0.226 – 0.228]	-0.023 [-0.025 - -0.022]	0.071 [0.070 - 0.073]
Females	0.204 [0.203 – 0.205]	0.065 [0.063 -0.066]	-0.002 [-0.003 - -0.001]
3 SACCADES			
Males	0.254 [0.253 - 0.255]	-0.206 [-0.208 - -0.204]	0.247 [0.245 - 0.249]
Females	0.299 [0.297 - 0.301]	-0.114 [-0.117 - -0.111]	0.184 [0.182 - 0.187]
4 ACTION OBSERVATION			
Males	0.317 [0.316 -0.318]	-0.292 [-0.297 - -0.288]	0.452 [0.450 - 0.454]
Females	0.314 [0.312 - 0.316]	-0.250 [-0.253 - -0.246]	0.411 [0.409 - 0.412]
5 DIVIDED ATTENTION (LEFT)			
Males	0.279 [0.277 - 0.280]	-0.186 [-0.189 - -0.183]	0.304 [0.302 - 0.306]
Females	0.304 [0.302 - 0.306]	-0.093 [-0.097 - -0.090]	0.251 [0.249 - 0.253]
6 DIVIDED ATTENTION (RIGHT)			
Males	0.278 [0.277 - 0.280]	-0.070 [-0.073 - -0.067]	0.252 [0.250 - 0.253]
Females	0.305 [0.302 - 0.308]	0.019 [0.016 - 0.022]	0.154 [0.152 - 0.156]
7 NARRATIVE			
Males	0.282 [0.281 - 0.284]	-0.185 [-0.189 - -0.182]	0.269 [0.267 - 0.270]
Females	0.269 [0.268 - 0.271]	-0.137 [-0.141 - -0.133]	0.248 [0.246 - 0.250]
8 WORD COMPREHENSION			
Males	0.300 [0.299 - 0.301]	-0.113 [-0.116 - -0.109]	0.210 [0.208 - 0.212]
Females	0.301 [0.300 - 0.303]	0.012 [0.008 - 0.016]	0.107 [0.104 - 0.110]
9 VERBAL FLUENCY			
Males	0.298 [0.297 - 0.300]	-0.099 [-0.104 - -0.095]	0.299 [0.296 - 0.301]
Females	0.331 [0.328 - 0.333]	-0.013 [-0.017 - -0.009]	0.176 [0.174 - 0.179]
10 AUTOBIOGRAPHICAL RECALL			
Males	0.326 [0.324 - 0.328]	-0.214 [-0.219 - -0.209]	0.408 [0.404 - 0.411]
Females	0.342 [0.340 - 0.345]	-0.067 [-0.073 - -0.062]	0.270 [0.267 - 0.274]

Supplementary Table 4: Mean standardized age β coefficients (slopes) and 95% *confidence interval* (CI) of the mean for all functional ROIs stratified by sex. While all slopes are significantly different from 0, some effects (standardized coefficients) are very small (e.g. GMD hand presses).

Functional region	Anatomical location of centroid
1 left-hand presses	Lobule V (left)
2 right-hand presses	Lobule V (right)
3 saccades	Lobule VIIIA (vermal)
4 action observation	Lobule IX (left)
5 divided attention (left)	Lobule Crus I (left)
6 divided attention (right)	Lobule VI (right)
7 narrative	Lobule Crus II (left)
8 word comprehension	Lobule Crus II (right)
9 verbal fluency	Lobule Crus I (right)
10 autobiographical recall	Lobule VIIIB (left)

Supplementary Table 5**:** Anatomical location of centroids (center point of each of the 10 ROIs) of functional regions.

	SRS score				Square-root transformed SRS score				
ROI	Estimate	*SE*	*p*-value	*FDR* corrected *p*-value	Estimate	*SE*	*p*-value	*FDR* corrected *p*-value	R^2 adjusted
Vermis I & II	-0.223	0.220	0.309	0.994	0.055	0.217	0.799	0.874	0.001
Lobule III (left)	-0.199	0.214	0.353	0.994	-0.007	0.211	0.973	0.973	0.002
Lobule IV (left)	-0.261	0.214	0.223	0.994	0.040	0.211	0.851	0.903	0.002
Lobule V (left)	-0.005	0.217	0.982	0.994	-0.242	0.214	0.258	0.437	0.003
Lobule VI (left)	-0.115	0.220	0.600	0.994	-0.217	0.217	0.317	0.462	0.005
Lobule Crus 1 (left)	-0.089	0.218	0.682	0.994	-0.283	0.215	0.188	0.391	0.007
Lobule Crus 2 (left)	0.128	0.214	0.551	0.994	-0.412	0.211	0.052	0.208	0.004
Lobule VIIB (left)	0.229	0.213	0.281	0.994	-0.650	0.210	0.002*	0.068	0.011
Lobule VIIIA (left)	0.141	0.214	0.511	0.994	-0.602	0.211	0.004*	0.077	0.013
Lobule VIIIB (left)	0.006	0.222	0.979	0.994	-0.311	0.219	0.156	0.389	0.004
Lobule IX (left)	-0.187	0.212	0.379	0.994	-0.106	0.210	0.613	0.716	0.004
Lobule X (left)	0.087	0.220	0.692	0.994	-0.371	0.217	0.087	0.277	0.004
Corpus medullare (left)	-0.050	0.216	0.816	0.994	-0.184	0.213	0.388	0.543	0.002
Vermis III	-0.188	0.214	0.379	0.994	0.067	0.211	0.752	0.849	0.000
Vermis IV	0.202	0.222	0.363	0.994	-0.421	0.219	0.054	0.208	0.003
Vermis V	-0.180	0.217	0.408	0.994	0.127	0.214	0.552	0.666	-0.001
Vermis VI	-0.002	0.223	0.994	0.994	-0.221	0.220	0.316	0.462	0.002
Vermis VIIA	0.330	0.224	0.141	0.994	-0.440	0.221	0.047	0.208	0.001
Vermis VIIB	0.212	0.221	0.337	0.994	-0.339	0.218	0.121	0.352	0.001
Vermis VIIIA	-0.080	0.215	0.712	0.994	-0.317	0.212	0.136	0.365	0.008
Vermis VIIIB	0.044	0.214	0.837	0.994	-0.459	0.211	0.030*	0.208	0.009
Vermis IX	-0.051	0.215	0.813	0.994	-0.270	0.212	0.202	0.393	0.005
Vermis X	-0.147	0.218	0.502	0.994	-0.011	0.215	0.959	0.973	0.000
Lobule III (right)	-0.013	0.214	0.953	0.994	-0.216	0.211	0.308	0.462	0.002
Lobule IV (right)	-0.068	0.221	0.759	0.994	-0.174	0.218	0.425	0.572	0.002
Lobule V (right)	-0.147	0.220	0.504	0.994	-0.141	0.217	0.515	0.654	0.003
Lobule VI (right)	-0.075	0.218	0.732	0.994	-0.282	0.215	0.190	0.391	0.006
Lobule Crus 1 (right)	0.016	0.213	0.939	0.994	-0.281	0.210	0.182	0.391	0.003
Lobule Crus 2 (right)	0.248	0.213	0.244	0.994	-0.527	0.210	0.012*	0.142	0.005
Lobule VIIB (right)	0.028	0.217	0.899	0.994	-0.454	0.214	0.034*	0.208	0.010
Lobule VIIIA (right)	-0.071	0.215	0.741	0.994	-0.400	0.212	0.059	0.208	0.012
Lobule VIIIB (right)	0.027	0.220	0.901	0.994	-0.418	0.217	0.055	0.208	0.008
Lobule IX (right)	-0.215	0.217	0.323	0.994	-0.137	0.214	0.524	0.654	0.006
Lobule X (right)	-0.080	0.221	0.717	0.994	-0.261	0.218	0.231	0.426	0.005
Corpus medullare (right)	-0.058	0.218	0.791	0.994	-0.241	0.215	0.262	0.437	0.004
left hand presses (VOL)	-0.145	0.217	0.503	0.994	-0.240	0.214	0.262	0.576	0.007
right hand presses (VOL)	-0.110	0.217	0.612	0.994	-0.237	0.214	0.269	0.576	0.006
saccades (VOL)	-0.123	0.222	0.580	0.994	-0.264	0.219	0.229	0.576	0.007
action observation (VOL)	0.041	0.214	0.849	0.994	-0.516	0.211	0.014*	0.164	0.013
divided attention (left) (VOL)	0.023	0.218	0.915	0.994	-0.382	0.215	0.076	0.327	0.006
divided attention (right) (VOL)	-0.002	0.216	0.994	0.994	-0.392	0.213	0.066	0.327	0.008
narrative (VOL)	-0.069	0.214	0.748	0.994	-0.196	0.212	0.355	0.592	0.003
word comprehension (VOL)	0.200	0.215	0.351	0.994	-0.509	0.212	0.016*	0.164	0.006
verbal fluency (VOL)	0.041	0.216	0.850	0.994	-0.375	0.213	0.078	0.327	0.006
autobiographical recall (VOL)	0.146	0.215	0.496	0.994	-0.577	0.212	0.007*	0.164	0.011
left hand presses (GMD)	-0.105	0.217	0.630	0.994	-0.088	0.214	0.682	0.854	0.001
right hand presses (GMD)	-0.150	0.215	0.483	0.994	0.052	0.212	0.804	0.909	0.000
saccades (GMD)	-0.066	0.222	0.768	0.994	0.009	0.219	0.966	0.966	-0.001
action observation (GMD)	-0.011	0.224	0.961	0.994	-0.187	0.220	0.396	0.626	0.001
divided attention (left) (GMD)	0.027	0.219	0.902	0.994	-0.223	0.216	0.302	0.582	0.001
divided attention (right) (GMD)	-0.122	0.217	0.572	0.994	-0.016	0.214	0.942	0.966	0.000
narrative (GMD)	0.041	0.222	0.853	0.994	-0.075	0.219	0.734	0.880	-0.001
word comprehension (GMD)	0.234	0.223	0.294	0.994	-0.254	0.220	0.248	0.576	0.000
verbal fluency (GMD)	-0.024	0.221	0.913	0.994	-0.089	0.218	0.683	0.854	0.000
autobiographical recall (GMD)	0.078	0.229	0.734	0.994	-0.217	0.225	0.335	0.591	0.000
left hand presses (WMD)	0.052	0.220	0.814	0.994	0.147	0.217	0.499	0.712	0.001
right hand presses (WMD)	0.052	0.218	0.812	0.994	0.037	0.215	0.864	0.926	0.000
saccades (WMD)	-0.004	0.220	0.986	0.994	0.050	0.217	0.819	0.909	-0.001
action observation (WMD)	-0.149	0.221	0.501	0.994	0.368	0.218	0.092	0.327	0.002
divided attention (left) (WMD)	-0.152	0.220	0.490	0.994	0.359	0.217	0.098	0.327	0.002
divided attention (right) (WMD)	0.021	0.220	0.924	0.994	0.156	0.217	0.474	0.711	0.001
narrative (WMD)	-0.081	0.222	0.716	0.994	0.125	0.219	0.570	0.777	-0.001
word comprehension (WMD)	-0.241	0.224	0.281	0.994	0.282	0.221	0.201	0.576	0.000
verbal fluency (WMD)	-0.069	0.225	0.761	0.994	0.225	0.222	0.310	0.582	0.001
autobiographical recall (WMD)	-0.297	0.225	0.186	0.994	0.419	0.222	0.059	0.327	0.001

Supplementary Table 6: Linear regression results investigating the effect of *Social Responsiveness Scale* (SRS) score (continuous) on deviation scores. SRS scores and square-root transformed SRS scores (given the skewed distribution, see Supplementary

Figure 7) were used as the independent variables. To account for the multiple tests, we applied the *false discovery rate – Benjamini Hochberg* (FDR-BH) correction within each parcellation separately.
