# Supplementary material for: Population-wide cerebellar growth models of children and adolescents
Source: Nat Commun. 2024 Mar 18;15:2351. doi: 10.1038/s41467-024-46398-2 (PMC10948906; doi:10.1038/s41467-024-46398-2)
Supplement: Supplementary file 3 — Reporting Summary [file 41467_2024_46398_MOESM3_ESM.pdf]

nature portfolio

Corresponding author(s): Maarten Frens

Last updated by author(s): Jan 25, 2024

Reporting Summary

Nature Portfolio wishes to improve the reproducibility of the work that we publish. This form provides structure for consistency and transparency in reporting. For further information on Nature Portfolio policies, see our Editorial Policies and the Editorial Policy Checklist.

Statistics

For all statistical analyses, confirm that the following items are present in the figure legend, table legend, main text, or Methods section.

n/a	Confirmed	
☐	☒	The exact sample size (*n*) for each experimental group/condition, given as a discrete number and unit of measurement
☐	☒	A statement on whether measurements were taken from distinct samples or whether the same sample was measured repeatedly
☐	☒	The statistical test(s) used AND whether they are one- or two-sided<br>*Only common tests should be described solely by name; describe more complex techniques in the Methods section.*
☐	☒	A description of all covariates tested
☐	☒	A description of any assumptions or corrections, such as tests of normality and adjustment for multiple comparisons
☐	☒	A full description of the statistical parameters including central tendency (e.g. means) or other basic estimates (e.g. regression coefficient) AND variation (e.g. standard deviation) or associated estimates of uncertainty (e.g. confidence intervals)
☐	☒	For null hypothesis testing, the test statistic (e.g. *F*, *t*, *r*) with confidence intervals, effect sizes, degrees of freedom and *P* value noted<br>*Give P values as exact values whenever suitable.*
☐	☒	For Bayesian analysis, information on the choice of priors and Markov chain Monte Carlo settings
☐	☒	For hierarchical and complex designs, identification of the appropriate level for tests and full reporting of outcomes
☐	☒	Estimates of effect sizes (e.g. Cohen's *d*, Pearson's *r*), indicating how they were calculated

Our web collection on statistics for biologists contains articles on many of the points above.

Software and code

Policy information about availability of computer code

Data collection	GE LX MR scanner software (release DV20.0-24.0) was used for data collection. XNET (version 1.7.4.1) was used for storage.
Data analysis	SMRIPrep (version: 20.1.1) was used for MRI preprocessing. Images were registered to standard stereotactic space (MN1152 NLin2009cAsym) using ANTs (version: 2.3.5). Images were segmented into anatomical ROIs using MAGeTBrain (version: 0.3.1.1) and into functional ROIs using the SUIT toolbox (version: 3.4) for Matlab. Normative models were generated using the PCNtoolbox (version: 0.27) running on pymc3 (version: 3.9.2). Custom-made Matlab (version: R2021b) and Python (version: 3.10.6) code used for statistical analysis and plotting are available upon request. Code to generate normative models and transfer knowledge from existing models to new sites is freely available via the PCNtoolkit (https://github.com/amarquand/PCNtoolkit) and the cerebellar growth models have been deposited on github (https://github.com/cgaiser1/cerebellar-growth-models).

For manuscripts utilizing custom algorithms or software that are central to the research but not yet described in published literature, software must be made available to editors and reviewers. We strongly encourage code deposition in a community repository (e.g. GitHub). See the Nature Portfolio guidelines for submitting code & software for further information.

Data

Policy information about availability of data

All manuscripts must include a data availability statement. This statement should provide the following information, where applicable:

- Accession codes, unique identifiers, or web links for publicly available datasets
- A description of any restrictions on data availability
- For clinical datasets or third party data, please ensure that the statement adheres to our policy

The cerebellar anatomical (https://github.com/CoBrALab/atlases) and functional (https://github.com/DiedrichsenLab/cerebellar_atlases) atlases are available on github. The cerebellar growth models have been deposited on github (https://github.com/cgaiser1/cerebellar-growth-models) as well. The raw MRI and participant-level region-of-interest data are protected and are not available due to privacy laws. However, access can be requested via the Generation R administration (secretariaat.genr@erasmusmc.nl). Source data are provided with this paper.

Research involving human participants, their data, or biological material

Policy information about studies with human participants or human data. See also policy information about sex, gender (identity/presentation), and sexual orientation and race, ethnicity and racism.

Reporting on sex and gender	Growth models of the cerebellum were stratified by sex (biological attribute), determined based on birth records. Data of 4,862 participants was available for statistical analysis, among which 2,470 were female and 2,392 male. Deviation scores of cerebellar growth were not stratified, because deviation scores were corrected for sex effects.<br>Gender (shaped by social and cultural circumstances) difference were not considered.
Reporting on race, ethnicity, or other socially relevant groupings	To illustrate clinically significant deviations we stratified participants by their scores on the Social Responsiveness Scale (threshold raw score > 90th percentile of cohort scores). The Social Responsiveness Scale has been shown to quantitatively assess subclinical and clinical autistic traits (Constantino et al., 2003) and was administered via questionnaire at the age of 8. Loss-of-follow up was assessed in terms of sex, parental national origin (Dutch, Non-Dutch but European, Non-European; obtained from birth records) household net income (low = < 1,20O€, middle = 1,200€ - 3,200€, high = >3,200€; obtained from<br>questionnaire), maternal education (higher education pursued or not; obtained from questionnaire), IQ and behavioral problems. Nonverbal IQ scores, normalized for sex and age, were measured using 2 subsets (Mosaics [spatial visualization] and Categories [abstract reasoning]) of the Snijders-Oomen Nonverbal Intelligence Test (SON-IQ) in the first measurement wave (mean age = 7.9). Behavioral problems were measured using the Child Behavioral Checklist (CBCL) (Achenbach, 2001) in the second measurement wave (mean age = 10.1). We dichotomized behavioral problems according to maternal reports (scoring above 80th percentile: behavioral problems present; below 80th percentile: behavioral problems not present).
Population characteristics	Participants characteristics for the main analyses included 1) age in years (mean: 11.2, range: 6,1 - 17,1) and 2) sex (2,470 (50.8%) female and 2,392 (49.2%) male).
Recruitment	Data comes from the Generation R Study, a large, prospective population-wide birth-cohort in Rotterdam, The Netherlands. Nearly 10,000 pregnant mothers were recruited between 2002 and 2006 with repeated measurements in the children and their parents over time. Since the cohort is based in the Netherlands, normative models might not generalize ideally to other populations. Participants from Western, educated, industrialized, rich, and democratic (WEIRD) societies are often overrepresented in research samples, particularly in small studies (Henrich et al., 2010). Yet, importantly the Generation R study is a population-based multi-ethnic cohort (Kooijman et al., 2016) and in the current study sample 29.8% of participants come from non-European backgrounds. Furthermore, the distribution of IQ scores in the current sample closely follows the population distribution (mean±std = 102.3±14.8). Additionally, the current models can easily be updated within the PCNtoolkit framework by including new data points outside of our age range and from diverse backgrounds, including different populations and clinical phenotypes.<br>Participants did not receive monetary compensation, but their travel costs were reimbursed. Additionally, as a token of appreciation for their participation, they received small gifts valued at 10€ or less, such as a drinking bottle, a bag, a power bank, or similar items. Written informed consent from both parents and assent from all participants was obtained.
Ethics oversight	Medical Ethical Committee of the Erasmus Medical Center

Note that full information on the approval of the study protocol must also be provided in the manuscript.

Field-specific reporting

Please select the one below that is the best fit for your research. If you are not sure, read the appropriate sections before making your selection.

☒ Life sciences ☐ Behavioural & social sciences ☐ Ecological, evolutionary & environmental sciences

For a reference copy of the document with all sections, see nature.com/documents/nr-reporting-summary-flat.pdf

Life sciences study design

All studies must disclose on these points even when the disclosure is negative.

Sample size	All participants with available MRI data from the prospective population-wide birth-cohort, the Generation R study, were included in the study, no prior sample size calculation was performed.
Data exclusions	Scans with insufficient quality ratings (i.e., cases without full coverage of the cerebellum, scans with substantial artifacts, and/or scans with marked inaccuracies in the parcellation) were excluded from analysis.
Replication	For both the anatomical and functional parcellations, we split the dataset into a training set (50%) and test set (50%) using the sex and scanner site variables to ensure equal distribution of sex and both scanners in both sets.
Randomization	Participants were not assigned to experimental conditions.
Blinding	Participants were not assigned to experimental conditions.

Reporting for specific materials, systems and methods

We require information from authors about some types of materials, experimental systems and methods used in many studies. Here, indicate whether each material, system or method listed is relevant to your study. If you are not sure if a list item applies to your research, read the appropriate section before selecting a response.

Materials & experimental systems

n/a	Involved in the study
☒	☐ Antibodies
☒	☐ Eukaryotic cell lines
☒	☐ Palaeontology and archaeology
☒	☐ Animals and other organisms
☒	☐ Clinical data
☒	☐ Dual use research of concern
☒	☐ Plants

Methods

n/a	Involved in the study
☒	☐ ChIP-seq
☒	☐ Flow cytometry
☐	☒ MRI-based neuroimaging

Plants

Seed stocks	*Report on the source of all seed stocks or other plant material used. If applicable, state the seed stock centre and catalogue number. If plant specimens were collected from the field, describe the collection location, date and sampling procedures.*
Novel plant genotypes	*Describe the methods by which all novel plant genotypes were produced. This includes those generated by transgenic approaches, gene editing, chemical/radiation-based mutagenesis and hybridization. For transgenic lines, describe the transformation method, the number of independent lines analyzed and the generation upon which experiments were performed. For gene-edited lines, describe the editor used, the endogenous sequence targeted for editing, the targeting guide RNA sequence (if applicable) and how the editor was applied.*
Authentication	*Describe any authentication procedures for each seed stock used or novel genotype generated. Describe any experiments used to assess the effect of a mutation and, where applicable, how potential secondary effects (e.g. second site T-DNA insertions, mosiacism, off-target gene editing) were examined.*

Magnetic resonance imaging

Experimental design

Design type	Not applicable.
Design specifications	Not applicable.
Behavioral performance measures	Not applicable.

Acquisition

Imaging type(s)	Structural
Field strength	3 Tesla
Sequence & imaging parameters	T1- weighted MRI scans were acquired using an inversion recovery fast spoiled gradient recalled sequence (IR-FSPGR)

using the following parameters: Wave 1: TR=10.3 ms, TE=4.2 ms, TI=350 ms, flip angle = 16°, acquisition time = 5 min 40 s, field of view = 230.4 x 230.4 mm, 0.9x0.9x0.9 mm3 isotropic resolution. Wave 2 and 3: TR=8.77 ms, TE=3.4 ms , TI=600 ms , flip angle = 10°, acquisition time = 5 min 20 s, field of view = 220 x 220 mm, lx1x1 mm3 isotropic resolution.

Area of acquisition: Whole brain

Diffusion MRI: ☐ Used ☒ Not used

Preprocessing

Preprocessing software	SMRIPrep (version: 20.1.1) was used for MRI preprocessing.
Normalization	Images were nonlinearly registered to standard stereotactic space using ANTs (version: 2.3.5) within SMRIPrep.
Normalization template	MNI152 NLin2009cAsym 1x1x1 mm resolution
Noise and artifact removal	Images were visually inspected and were excluded if substantial artifacts, and/or inaccuracies in the parcellation were present.
Volume censoring	Not applicable.

Statistical modeling & inference

Model type and settings	Normative modeling using Bayesian Hierarchical regression to predict cerebellar ROIs (volume, GMD, WMD) from the covariates: age, sex, & scanner. We employed a sinh-arcsinh likelihood (SHASHb) to accommodate non-Gaussian distributions and modeled random effects in intercept, slope, and variance on the batch-effects (sex and scanner). Regression models for each region of interest (ROI) were generated separately.
Effect(s) tested	Model outputs include the posterior distributions of the parameters and deviations from the normative range (z-score which are free of batch-effects) for each individual in the test set. Model performance was evaluated using Leave-one-out crossvalidation (LOO).

Specify type of analysis: ☐ Whole brain ☒ ROI-based ☐ Both

Anatomical location(s): Anatomical ROIs = MAGeTBrain, Park et al. 2014
Functional ROIs = MDTB atlas, King et al., 2019

Statistic type for inference (See Eklund et al. 2016)	Structural ROI-level inference.
Correction	Not applicable.

Models & analysis

n/a	Involved in the study
☒	☐ Functional and/or effective connectivity
☒	☐ Graph analysis
☐	☒ Multivariate modeling or predictive analysis

Multivariate modeling and predictive analysis: Normative modeling in Bayesian Hierarchical regression framework. Independent variables: age, sex, & scanner; dependent: Volume/GMD/WMD in cerebellar ROIs. Training/Test split 50%/50% ensuring equal age, sex, and scanner distribution in both training and test sets. Evaluation metric: Leave-one-out cross-validation (LOO) and posterior distributions (mean and confidence interval) of parameters.
